# Phytolith‐occluded carbon sequestration potential in three major steppe types along a precipitation gradient in Northern China

**DOI:** 10.1002/ece3.7155

**Published:** 2021-01-04

**Authors:** Limin Qi, Tingyu Sun, Xudong Guo, Ying Guo, Frank Yonghong Li

**Affiliations:** ^1^ Ministry of Education Key Laboratory of Ecology and Resource Use of the Mongolian Plateau & Inner Mongolia Key Laboratory of Grassland Ecology School of Ecology and Environment Inner Mongolia University Hohhot China; ^2^ Collaborative Innovation Center for Grassland Ecological Security Ministry of Education of China and Inner Mongolia Autonomous Region Inner Mongolia University Hohhot China; ^3^ Institute of Grassland Research of Chinese Academy of Agricultural Sciences Hohhot China

**Keywords:** belowground PhytOC production, desert steppe, perennial grass, phytolith‐occluded carbon, species composition, typical steppe

## Abstract

Phytolith‐occluded carbon (PhytOC) is an important long‐term stable carbon fraction in grassland ecosystems and plays a promising role in global carbon sequestration. Determination of the PhytOC traits of different plants in major grassland types is crucial for precisely assessing their phytolith carbon sequestration potential. Precipitation is the predominant factor in controlling net primary productivity (NPP) and species composition of the semiarid steppe grasslands. We selected three representative steppe communities of the desert steppe, the dry typical steppe, and the wet typical steppe in Northern Grasslands of China along a precipitation gradient, to investigate their species composition, biomass production, and PhytOC content for quantifying its long‐term carbon sequestration potential. Our results showed that (a) the phytolith and PhytOC contents in plants differed significantly among species, with dominant grass and sedge species having relatively high contents, and the contents are significantly higher in the below‐ than the aboveground parts. (b) The phytolith contents of plant communities were 16.68, 17.94, and 15.85 g/kg in the above‐ and 86.44, 58.73, and 76.94 g/kg in the belowground biomass of the desert steppe, the dry typical steppe, and the wet typical steppe, respectively; and the PhytOC contents were 0.68, 0.48, and 0.59 g/kg in the above‐ and 1.11, 0.72, and 1.02 g/kg in the belowground biomass of the three steppe types. (c) Climatic factors affected phytolith and PhytOC production fluxes of steppe communities mainly through altering plant production, whereas their effects on phytolith and PhytOC contents were relatively small. Our study provides more evidence on the importance of incorporating belowground PhytOC production for estimating phytolith carbon sequestration potential and suggests it crucial to quantify belowground PhytOC production taking into account of plant perenniality and PhytOC deposition over multiple years.

## Graphical abstract

PhytOC stores of plants in various grassland steppe types show distinct difference. Climatic factors affected phytolith and PhytOC production of steppe communities mainly through altering plant production. The importance of the PhytOC store in belowground production should be taken into account when estimating PhytOC sequestration potential.

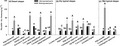

## 1 INTRODUCTION

Climate warming and the increase of extreme climatic events are mostly attributed to the increasing concentrations of greenhouse gases in the atmosphere, especially carbon dioxide (CO_2_). The coupled biogeochemical cycles of carbon (C) and silicon (Si) in the terrestrial system are deemed a mechanism that impacts the long‐term regulation of atmospheric CO_2_ (Berner, 1992; Parr & Sullivan, 2011; Parr et al., 2010; Song, Liu, et al., 2012; Song, Wang, et al., 2012). Plants absorb mono‐silicic acid from soil solution via their roots (Epstein, 1994; Ma & Yamaji, 2006) and deposit silica (SiO_2_) in plant tissues as phytoliths (Hodson et al., 2005; Schaller et al., 2013). Approximately 0.1%–6.0% organic C in plants is reported to be incorporated in phytoliths during their formation and is referred to as phytolith‐occluded carbon (PhytOC) (Bartoli and Wilding, 1980; Jones & Milne, 1965; Zuo & Lü, 2011). Phytoliths exist in most plants, and their content in plant tissues varies among plant species, ranging from less than 0.5% in most dicotyledons, 1%–3% in dryland grasses, and up to 10%–15% in the Cyperaceae and wetland Poaceae species (Epstein, 1994). Phytoliths are mainly deposited of the cell wall, cell lumen, and intercellular spaces or the extracellular layer in plant issues (Epstein, 2009; Hodson et al., 2005; Ma & Yamaji, 2006; Schaller et al., 2013); after plants' death, the phytoliths can be incorporated into soil or sediments after the decomposition of plant litter (Blecker et al., 2006). Since phytoliths are resistant to decomposition, PhytOC can be preserved in soil or sediments with the Si‐coat protection for several hundred or thousand years (Hodson, 2019; Song et al., 2016) and may account for 82% of the total organic carbon in some old soils (Parr & Sullivan, 2005; Song et al., 2017). The formation and stability of PhytOC in the coupled biogeochemical cycles of C and Si have been increasingly recognized as a promising mechanism of terrestrial ecosystems to sequester atmospheric CO_2_, which has motivated many researchers to quantify the PhytOC sequestration potential of various terrestrial systems (Parr & Sullivan, 2011; Parr et al., 2010; Song, Liu, et al., 2012).

Grasslands are an important terrestrial ecosystem covering more than one‐fifth of the world's land surface (Scurlock & Hall, 2010). The large distribution area and the high PhytOC concentration in grassland plants, especially Poaceae and Cyperaceae species (Clarkson & Hanson, 1980; Epstein, 1994), make grassland a particularly important long‐term C sequestration process (Blecker et al., 2006; Song et al., 2017). Several studies assessed the PhytOC sequestration potential of grasslands (Pan et al., 2017; Qi et al., 2016; Song, Liu, et al., 2012). Song, Liu, et al. (2012) reported that phytolith and PhytOC production rates in aboveground biomass of grassland were significantly influenced by their aboveground net primary productivity (ANPP); Qi et al. (2016) suggested that the belowground productivity of plants could play a dominant role in PhytOC production in grassland ecosystems; and Ji et al. (2018) reported that Si distribution in the aboveground parts, thus the PhytOC sequestration potential, of grassland plants, varies markedly among plant species and across grassland types. However, the effects of grassland type and species composition on the production rate of phytoliths and PhytOC (including the above‐ and belowground parts) are still not fully understood. Precipitation is the predominant climatic factor that controls plant species composition and net primary productivity (NPP) of grassland ecosystems in semiarid steppe region (Bai et al., 2004; Dai et al., 2012; Hou et al., 2014), thus affecting the PhytOC sequestration potential. As such, it is necessary to investigate the PhytOC production rate in different grassland types along a climatic gradient, for accurate estimation of PhytOC sequestration potential and prediction of their response to climate changes. In this study, we selected three representative vegetation types in climatically different regions along a precipitation gradient in Inner Mongolia, that is, the desert steppe, the dry typical steppe, and the wet typical steppe regions, to study their PhytOC production. Specifically, we aimed to investigate the species compositions of these plant communities, and measure the phytolith and PhytOC contents of the major plant species, of these grassland types, so as to increase the accuracy in estimating phytoliths and PhytOC productions and storages in different plant communities, and analyze their relations with climatic factors. We hypothesized that along the gradient of climate aridity increase (that is, precipitation decrease) from the wet typical steppe site, via the dry typical steppe site to the desert steppe site, the grassland NPP would decrease (Bai et al., 2008), while the contents of phytoliths and PhytOC in the steppe plants would increase due to the increase in plant transpiration intensity.

## 2 MATERIALS AND METHODS

### 2.1 Sampling sites

This study was conducted at three sites in the steppe region of central Inner Mongolia along a climatic gradient of increasing annual precipitation, that is, at a desert steppe site (within Sunite Right Banner, at 43°511′N, 113°42′E), a dry typical steppe site (within Maodeng farm of Xilinhot city, at 44°50′N, 116°36′E), and a wet typical steppe site (within West Ujimqin Banner, at 45°43′N, 118°30′E). The region experiences a temperate semiarid climate. The mean annual temperature (MAT) was 0.19, 2.67, and 3.32°, and mean annual precipitation (MAP) was 182, 278, and 342 mm, respectively, at the desert steppe, the dry typical steppe, and the wet typical steppe sites; and 75%–85% of annual precipitation falls in the plant growing seasons from May to September (average of the 1960–2016 period). In the year for field study (2016), the annual temperature (TEMP) were 0.98, 3.49, and 4.50°C and annual precipitation were 189, 309, and 299 mm, and the plant growing season precipitation was 129, 215, and 237 mm, respectively, in the desert steppe, the dry typical steppe, and the wet typical steppe sites. The desert steppe site is on a calcic brown soil, whereas the other two steppe sites are on chestnut soil. The humus layer is 15–30 cm in the soil profile, and the calcic horizon (mostly CaCO_3_) is 30–60 cm in the soil profile, both increasing from the desert steppe to the dry typical steppe and the wet typical steppe. The dominant species of the vegetation are *Stipa klemenzii, Cleistogenes songorica, Allium bidentatum,* and *Salsola collina* in the desert steppe, *Leymus chinensis, Stipa krylovii,* and *Cleistogenes squarrosa* in the dry typical steppe, and *Leymus chinensis, Stipa grandis,* and *Cleistogenes squarrosa* in the wet typical steppe (Table 2).

### 2.2 Field sampling

In each of the three steppe sites along the precipitation gradient, three delineated plots of 20 m × 20 m located in three separate farms were selected for plant and soil sampling. These plots were on flat ground and covered with representative native steppe communities and fenced to exclude animal grazing at the beginning of the plant growing season in 2016. Plant and soil samples were collected from these plots at the end of August. Five quadrates of 1 m × 1 m were set up at the center and four corners of each delineated plots, and all standing live and dead vascular plants (that was produced during the current season) in these quadrates were harvested at ground level species by species, dried to a constant weight at 65℃ and weighed. The dry mass of all plant species per quadrat averaged over five replicates was used to determine the aboveground plant biomass at peak plant biomass time, and this was also used to approximate ANPP of the grassland (Scurlock et al., 2002). The belowground biomass and its distribution profile (0–70 cm) were measured using the soil coring method, and BNPP (during the plant growing season from May to October) of the studied grassland was obtained from previous studies (Chai et al., 2014; Hou et al., 2014).

Whole plants of dominant species in each sampling plot (within a separate farm) were collected by digging up each individual to a depth of 20 cm below ground level, and then each individual was cut into two parts: aboveground part (shoots) and belowground part (noted as roots, but it includes plant roots and rhizomes as well as shoot stumps buried below the soil surface). A sample of about 300 g dry matter of the shoots and the roots of each plant species was collected in each of the three sampling plots at each site. The samples were washed with deionized water, dried at 65°C and then cut into pieces (<5 mm) for phytolith analysis.

The soil bulk density and moisture content of soil profile (0–70 cm) were obtained by the cutting‐ring method and the oven drying method (at 105°C). The soil samples were collected using soil cores (diameter = 7 cm) and air dried at ambient temperature in the laboratory.

### 2.3 Sample analysis

The phytoliths within plant parts were extracted using a microwave digestion process (Parr et al., 2001) followed by a Walk‐Black type digestion to ensure the purity of the phytoliths (Parr & Sullivan, 2014; Walkley & Black, 1934). Two duplicates were analyzed for each plant sample. The exacted phytoliths were dried at 65°C to a constant weight. The PhytOC was determined using the PhytOC alkalidis solution spectrophotometer method (Yang et al., 2014). In this method, sodium hydroxide solution was used to dissolve the Si compound in order to release the occluded organic carbon from the phytoliths, then potassium dichromate (K_2_Cr_2_O_7_)‐sulfuric acid (H_2_SO_4_) solution was used to oxidize the released organic carbon, and the concentration of Gr^3+^ produced in this oxidation was determined by spectrophotometer with its absorbance at 590 mm wavelength. The organic carbon concentration was calculated based on the amounts of potassium dichromate consumed, and the accuracy and repeatability of the method were well verified against the results obtained with acid dissolution‐Elementar Vario MAX CN method (Germany) (Yang et al., 2014). The phytoliths and PhytOC contents of the two parts of each plant species were calculated as the average of the three replicate plots. For each species, the ratio of aboveground to belowground biomass (shoots/roots) was calculated based on the sampled plant individuals. The ratio was used to calculate the belowground biomass of the species in 1 m^2^ based on the measured aboveground biomass of the species.

The air‐dried soil samples were separated into the 100 mesh soil samples and the 10 mesh soil samples. The soil organic carbon (SOC) was determined with the 100 mesh soil samples using the method of classical potassium dichromate (Walkley & Black, 1934), and the soil pH and bioavailable Si content were analyzed with 10 mesh soil samples using a pH meter and silicomolybdic acid method (Yang et al., 2018), respectively.

### 2.4 Data calculations and statistics

The formulae for calculating phytolith and PhytOC contents in plant species were as follows:

(1) phytolithcontent(g/kg)=phytolithweight(g)/drybiomass(kg)

(2) PhytOCcontent(g/kg)=PhytOCweight(g)/drybiomass(kg)

The community‐weighted mean contents of phytolith (Phytolith_CWM_) and PhytOC (PhytOC_CWM_) were also calculated using the contents of phytolith and PhytOC in the species of the community and the relative biomass of the species (as weight), similar to the calculation of other community‐weighted mean plant traits (Ricotta & Moretti, 2011).

(3) $${\text{phytolith}}_{\text{CWM}}\left(g/\text{kg}\right)=\sum_{i}{\text{phytolith content}}_{i}\left(g/\text{kg}\right)\times {\text{biomass}}_{i}\left(\%\right)$$

(4) $${\text{PhytOC}}_{\text{CWM}}\;\left(g/\text{kg}\right)=\sum_{i}\text{PhytOC}\;{\text{content}}_{i}\left(g/\text{kg}\right)\times {\text{biomass}}_{i}\;\left(\%\right)$$

where *i* enumerates each species.

(5) $$\text{PhytOC}\;\text{stock}\;\left(\text{kg}/\text{ha}\right)={\text{PhytOC}}_{\text{CWM}}\left(g/\text{kg}\right)\times \text{biomass}\;\left(\text{kg}/\text{ha}\right)\times 10^{‐3}$$

(6) $$\text{PhytOC}\;\text{production}\;\text{flux}\;\left(\text{kg}\;{\text{ha}}^{‐1}\;{\text{year}}^{‐1}\right)={\text{PhytOC}}_{\text{CWM}}\left(g/\text{kg}\right)\times \text{NPP}\;\left(\text{kg}\;{\text{ha}}^{‐1}\;{\text{year}}^{‐1}\right)\times 10^{‐3}$$

One‐way ANOVA and Duncan's multiple range test were performed to examine the difference in phytolith and PhytOC contents among different parts of plant species. A principal component analysis (PCA) of PhytOC content and production parameters and environmental factors were performed to show their interrelations. R 3.3.3 was used for all the statistics, and Sigma Plot 12.0 was used for figures.

## 3 RESULTS

### 3.1 Soil properties at the three steppe sites

Soil physical and chemical properties differed among the three sites and there were significant differences in soil bulk density, moisture, pH, and SOC content (Table 1). Soil bulk density was higher in the wet typical steppe than other steppes (*p* < .05). Soil moisture and the SOC content increased, while soil pH decreased, from the desert steppe to the dry typical steppe, and the wet typical steppe (*p* < .05). The bioavailable Si content in soil showed no significant difference among the three sites.

**TABLE 1:** The soil bulk density (BD), moisture, pH, organic carbon (SOC), and bioavailable Si contents at the three steppe sites, and analysis of variance of these soil indicators (*F*‐values and *p*‐values) across the sites

Sites	BD (g/cm^3^)	Moisture (%)	pH	SOC (g/kg)	Bioavailable Si (mg/kg)
Desert steppe	1.30b	2.79c	8.77a	0.90c	0.26a
Dry typical steppe	1.32b	14.71b	8.05a	2.81b	0.32a
Wet typical steppe	1.49a	21.32a	7.51b	3.12a	0.27a
ANOVA ‐*F*	57.89	348.3	6.381	49.14	0.978
ANOVA ‐*p*	<.001	<.001	<.001	<.001	.429

Note

Different letters indicate significant difference among steppe types at *p* < .05.

### 3.2 Plant above‐ and belowground biomass

Eight, seven, and three dominant species were sampled, respectively, in the desert steppe, the dry typical steppe and the wet typical steppe communities to determine their phytolith and PhytOC contents; these species contributed to 87.58%, 93.65%, and 94.39% of the aboveground biomass of the three plant communities (Table 2). The measured aboveground community biomass was 562.27 ± 49.28, 1,471.99 ± 110.82, and 1,120.09 ± 52.98 kg/ha, and the belowground biomass (0–70 cm) were 4,227.99 ± 456.20, 8,639.89 ± 543.77, and 8,643.74 ± 491.55 kg/ha in the desert steppe, the dry typical steppe, and the wet typical steppe, respectively. The biomass of these plant species in these communities was shown in Table 2.

**TABLE 2:** The aboveground (AGB) and belowground biomass (BGB) (kg/ha; mean ± *SE*) of the three major steppe communities and their major composing species in Inner Mongolia. (A/B refers to the AGB/BGB ratio)

Types	Species name	AGB	BGB	A/B
Desert steppe	*Stipa klemenzii*	256.89 ± 61.11	1,511.11 ± 359.45	0.17
	*Cleistogenes songorica*	83.00 ± 42.06	237.14 ± 120.17	0.35
	*Salsola collina*	73.22 ± 58.74	95.09 ± 76.28	18.23
	*Scorzonera divaricata*	21.67 ± 21.67	4.47 ± 4.47	4.85
	*Allium mongolicum*	18.11 ± 8.71	95.32 ± 45.82	0.19
	*Caragana pygmaea*	17.67 ± 15.71	0.97 ± 0.86	0.77
	*Carex korshinskyi*	13.78 ± 2.41	52.99 ± 9.25	0.26
	*Allium bidentatum*	8.44 ± 7.00	140.74 ± 116.64	0.06
	Sum of major species	492.78	2,137.83^a^	
	Community	562.27	4,227.99^a^	
	*Leymus chinensis*	404.68 ± 40.26	586.49 ± 132.52	0.69
	*Stipa krylovii*	394.12 ± 231.38	985.31 ± 227.18	0.40
	*Stipa grandis*	352.92 ± 175.27	840.29 ± 163.22	0.42
	*Cleistogenes squarrosa*	132.59 ± 62.40	301.34 ± 20.67	0.44
	*Agropyron michnoi*	72.67 ± 72.67	117.20 ± 18.54	0.62
	*Carex korshinskyi*	14.32 ± 14.32	95.48 ± 1.08	0.15
	*Artemisia frigida*	7.16 ± 7.16	3.44 ± 0.66	2.08
	Sum of major species	1,378.64	2,929.55	
	Community	1,471.99	8,639.89	
Wet typical steppe	*Stipa grandis*	553.48 ± 168.32	1,777.45 ± 393.71	0.31
	*Cleistogenes squarrosa*	303.37 ± 122.25	864.56 ± 316.41	0.33
	*Leymus chinensis*	200.39 ± 83.52	301.21 ± 113.58	0.67
	Sum of major species	1,057.24	2,943.22	
	Community	1,120.09	8,643.74	

^a^ The sum of the BGB of major species is the BGB in top 20 cm soil layer and the BGB of community of soil profile (0–70 cm) determined in this study.

### 3.3 Phytolith and PhytOC contents in grassland plants

**FIGURE 1:**
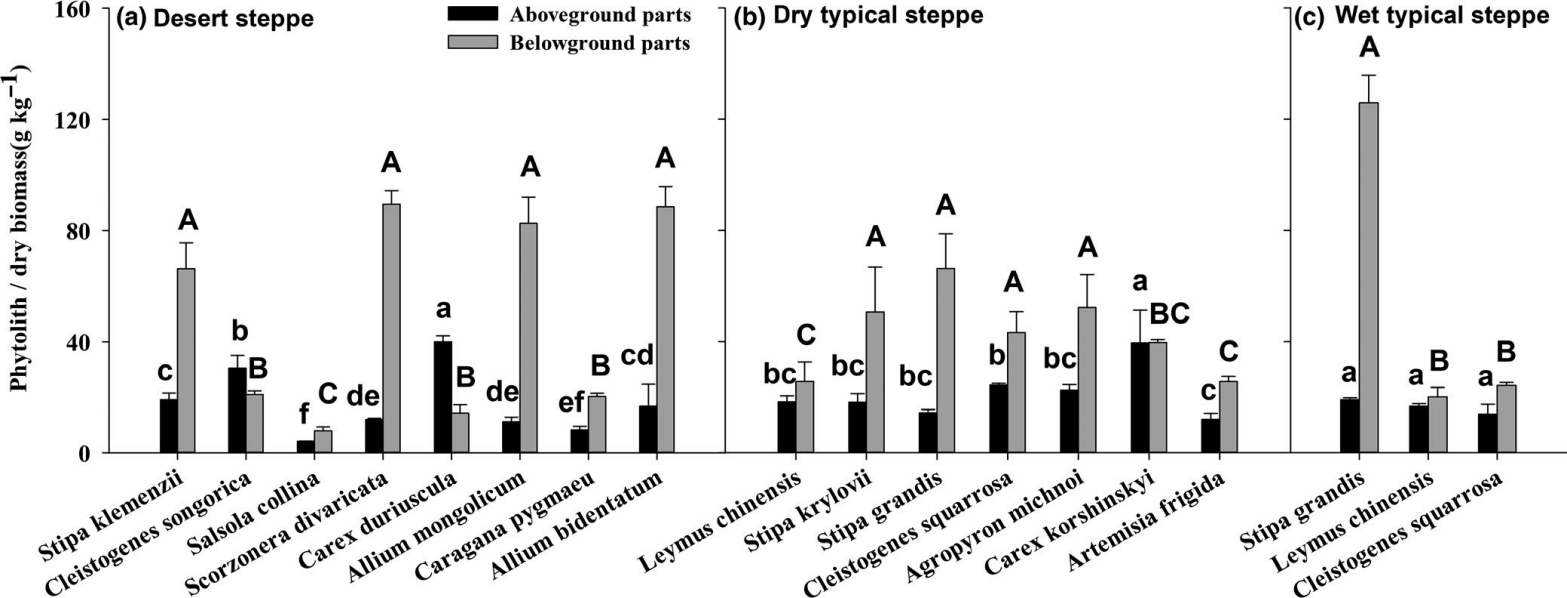
The phytolith content in the above‐ and belowground parts of dominant plants in the desert steppe (a), the dry typical steppe (b), and the wet typical steppe (c) in Inner Mongolia. Species are ordered according to their relative biomass in steppe communities. Different lowercase and uppercase letters indicate significant differences in the above‐ and the belowground plant parts among species, respectively (*p* < .05)

**FIGURE 2:**
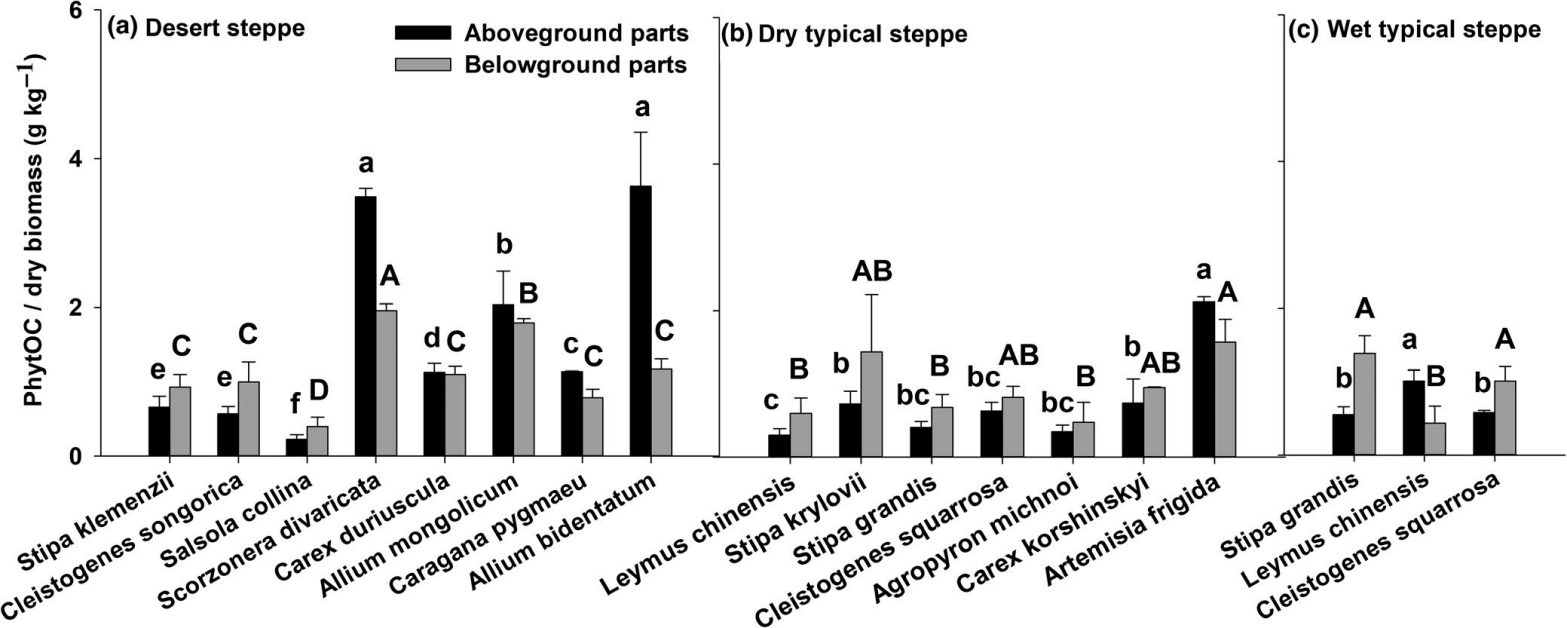
The PhytOC content in the above‐ and belowground parts of dominant plants in the desert steppe (a), the dry typical steppe (b), and the wet typical steppe (c) in Inner Mongolia. Species are ordered according to their relative biomass in steppe communities. Different lowercase and uppercase letters indicate significant differences in the above‐ and the belowground plant parts among species, respectively (*p* < .05)

Phytolith and PhytOC contents in the above‐ and belowground biomass varied substantially among plant species, and across grassland types (Table 3, Figures 1 and 2). In the aboveground parts, the phytolith content was highest in *Carex duriuscula* (39.96 ± 2.21 g/kg) and lowest in *Salsola collina* (4.23 ± 0.09 g/kg) in the desert steppe. The phytolith content in belowground biomass varied from 7.99 ± 1.34 g/kg in *Salsola collina* in the desert steppe to 126.0 ± 9.86 g/kg in *Stipa grandis* in the wet typical steppe. The PhytOC content in aboveground biomass was in the range of 0.23 ± 0.06 g/kg in *Salsola collina* to 3.63 ± 0.73 g/kg in *Allium bidentatum* in the desert steppe; the PhytOC content in belowground biomass was also lowest in *Salsola collina* (0.40 ± 0.13 g/kg), but highest in *Scorzonera divaricata* (1.96 ± 0.09 g/kg) in the desert steppe (Figure 2).

**TABLE 3:** One‐way ANOVA of phytolith and PhytOC contents in the aboveground (AGB) and belowground biomass (BGB) of steppe species and communities at the three steppe sites

Contents	Species in desert steppe			Species in dry typical steppe			Species in wet typical steppe			Communities at three sites		
	*df*	*F*	*p*	*df*	*F*	*p*	*df*	*F*	*p*	*df*	*F*	*p*
Phytolith content in AGB	7	43.60	<.001	6	6.434	<.001	2	1.434	.310	2	5.333	.467
Phytolith content in BGB	7	44.67	<.001	6	6.659	<.001	2	135.6	<.001	2	1.464	.303
PhytOC content in AGB	7	421.30	<.001	6	23.890	<.001	2	5.765	.0401	2	6.421	.323
PhytOC content in BGB	7	30.32	<.001	6	1.990	.135	2	6.194	.0347	2	1.525	.291

### 3.4 Community‐weighted mean contents of phytolith and PhytOC

The community‐weight mean phytolith content (phytolith_CWM_) and PhytOC content (PhytOC_CWM_) in the above‐ and belowground plant biomass were calculated. As plant belowground biomass was measured at species, not community level, the relative belowground biomass of each species was estimated using the measured relative aboveground biomass (AGB%) and the shoot/root ratio (A/B) of each species, and used to calculate the community‐weighted mean phytolith content and PhytOC content of belowground biomass.

**FIGURE 3:**
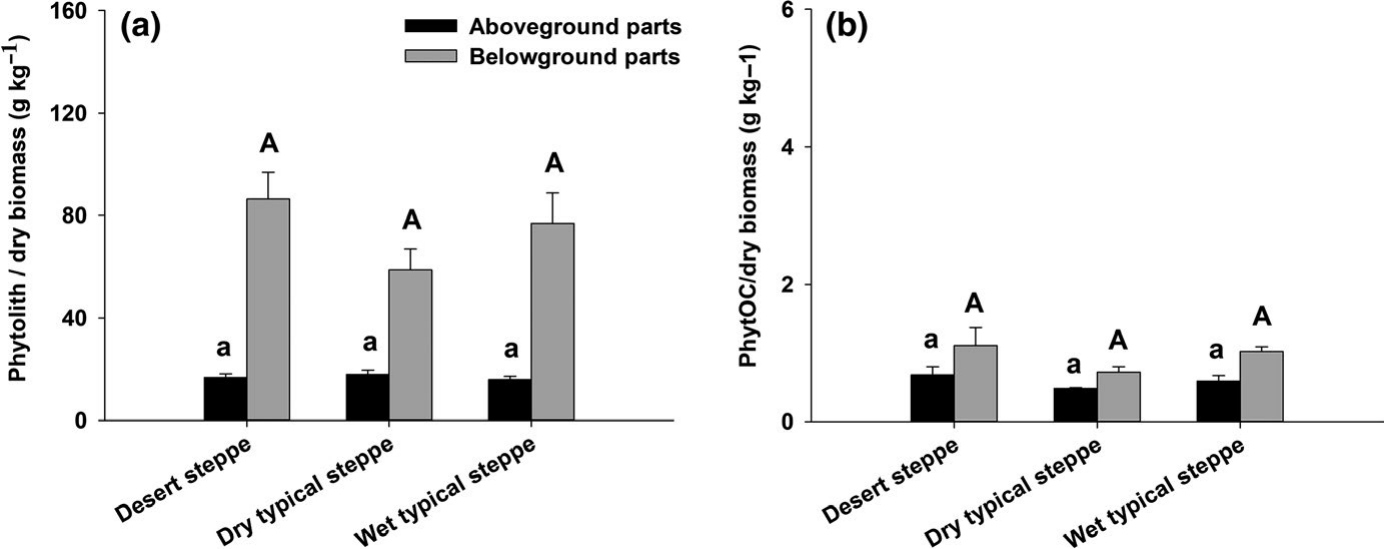
The phytolith content (a) and PhytOC content (b) in the above‐ and belowground parts of steppe communities in Inner Mongolia. No significant differences are detected for either above‐ or belowground parts among steppe types (*p* < .05)

The phytolith_CWM_ was 16.68, 17.94, and 15.85 g/kg, respectively, in the aboveground biomass of the desert steppe, the dry typical steppe, and the wet typical steppe, no significant difference being detected among the three steppe types (*p* > .05); the phytolith_CWM_ in the belowground biomass was neither significantly different among the three steppe types (*p* > .05), being 86.44, 58.73, and 76.94 g/kg for the desert steppe, the dry typical steppe and the wet typical steppe, respectively (Figure 3a). The PhytOC_CWM_ was 0.68 g/kg in the aboveground biomass of the desert steppe, which was higher than in that of the dry typical steppe (0.48 g/kg) or the wet typical steppe (0.59 g/kg). The PhytOC_CWM_ in the belowground biomass was 1.11, 0.72, and 1.02 g/kg, respectively, in the desert steppe, the dry typical steppe, and the wet typical steppe, with no significant difference among these steppes (*p* > .05) (Figure 3b).

### 3.5 The PhytOC sequestration potential and its relations with environmental factors

The PhytOC stock in the aboveground biomass ranged from 0.38 ± 0.07 to 0.71 ± 0.09 kg/ha and was significantly lower in the desert steppe than in two typical steppes (*p* < .05); and PhytOC stock in the belowground biomass ranged from 4.67 ± 0.16 to 8.79 ± 0.36 kg/ha and was significantly lower in the desert steppe and the dry typical steppe than in the wet typical steppe (*p* < .05) (Table 4).

**TABLE 4:** Estimation of plant aboveground biomass (AGB) and net primary productivity (ANPP), belowground biomass (BGB) and net primary productivity (BNPP), and biomass‐weighted community mean PhytOC content, stock, and production flux (mean ± *SE*) of the three steppe types in Inner Mongolia

Steppe types	PhytOC content (g/kg)		AGB = ANPP (kg ha^−1^ year^−1^)	BGB (0−70 cm)	BNPP* (kg ha^−1^ year^−1^)	PhytOC stock (kg/ha)		PhytOC production flux (kg ha^−1^ year^−1^)
	Aboveground	Belowground				Aboveground	Belowground	Belowground
Desert steppe	0.68 ± 0.12a	1.11 ± 0.26a	562.65 ± 49.28b	4,227.99 ± 456.20b	7,964	0.38 ± 0.07b	4.67 ± 0.16b	7.88
Dry typical steppe	0.48 ± 0.01a	0.72 ± 0.08a	1,471.99 ± 110.82a	8,639.89 ± 543.77a	13,446	0.71 ± 0.09a	6.20 ± 0.23ab	9.14
Wet typical steppe	0.59 ± 0.08a	1.02 ± 0.07a	1,120.09 ± 52.98a	8,643.74 ± 491.55a	17,955	0.66 ± 0.01a	8.79 ± 0.36a	21.01

Note

The range of BNPP determined in several studies on the Mongolian steppes (Chai et al., 2014; Hou et al., 2014) and the above‐ and belowground biomass (0–70 cm) determined in this experiment. Different lowercase letters indicate significant differences among steppe types at *p* < .05 (Duncan's test).

The PhytOC production flux of steppe communities was estimated based on the NPP and its PhytOC content, and the BNPP was quoted from previous results conducted on the studied grasslands (Chai et al., 2014; Hou et al., 2014). The PhytOC production flux of steppe communities were 0.38, 0.71, and 0.66 kg ha^−1^ year^−1^ from the aboveground parts (ANPP), and 7.88, 9.14, and 21.01 kg ha^−1^ year^−1^ from the belowground parts (BNPP), respectively, in the desert steppe, the dry typical steppe and the wet typical steppe (Table 4).

**FIGURE 4:**
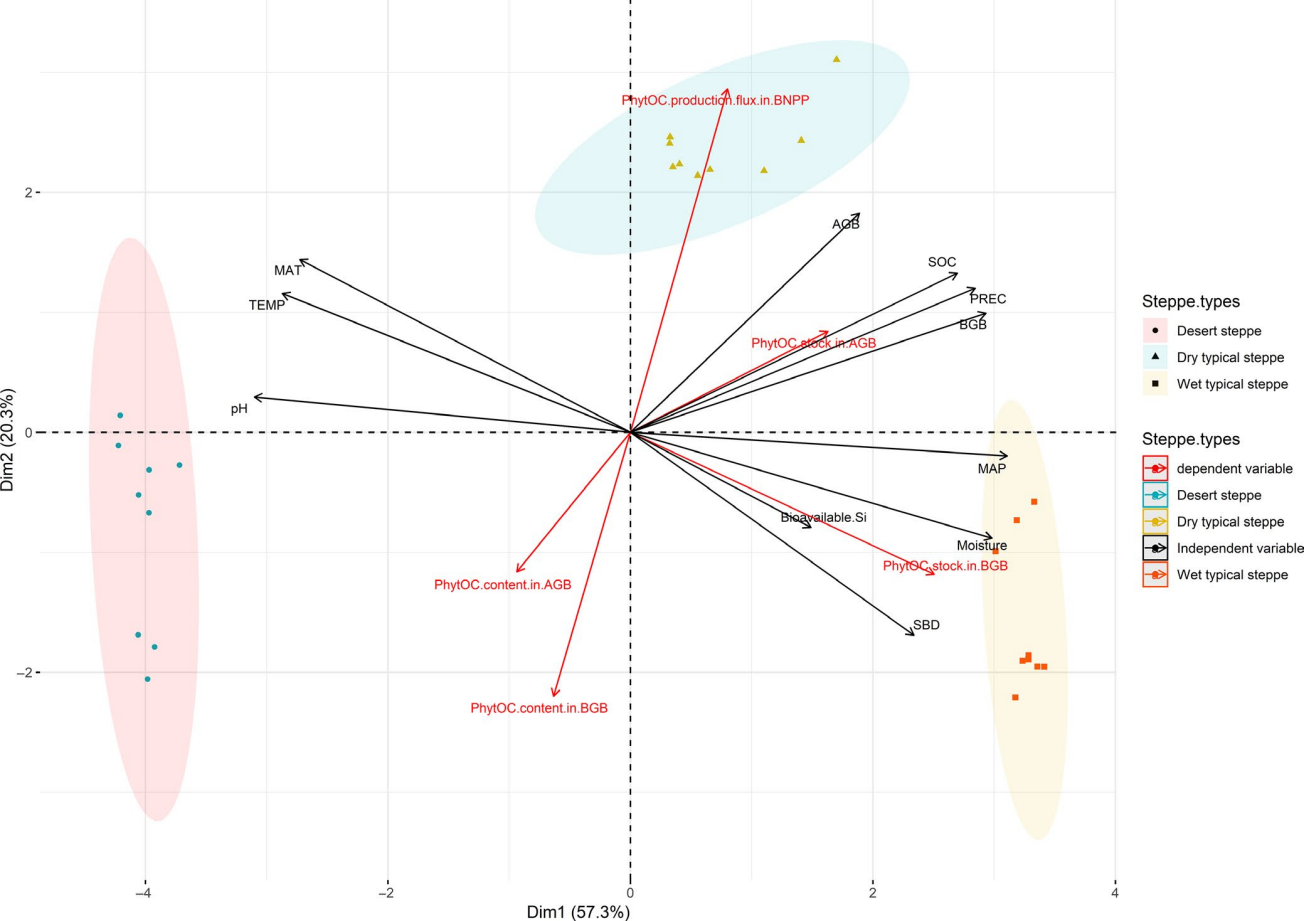
A principal component analysis showing the relationships among PhytOC production parameters (PhytOC content, stock, and production flux in above‐ or belowground biomass), plant above‐ and belowground biomass (AGB and BGB), and environmental factors of long‐term mean annual precipitation (MAP) and temperature (MAT), current‐year precipitation (PREC), and temperature (TEMP), soil bulk density (SDB), organic carbon content (SOC), bioavailable Si content, and pH

The PCA among the PhytOC production parameters and environmental factors (Figure 4) showed that PhytOC contents in both AGB and BGB had an opposite change trend to that of AGB and BGB, indicating the steppes with low biomass had relatively high PhytOC contents. However, PhytOC production flux in ANPP (i.e., PhytOC in AGB) and in BNPP showed the similar trend as that of AGB and BGB, suggesting the variation in PhytOC production flux being mainly associated with the variation in biomass production, not PhytOC content. Furthermore, PhytOC stock in BGB, and to some extent in AGB, showed the same trend as that of bioavailable Si content in soil; the PhytOC stock in BGB was significantly correlated with bioavailable Si content and with soil moisture (*p* < .01) (Figure 4).

## 4 DISCUSSION

### 4.1 Phytolith and PhytOC contents in grassland ecosystems

The dominant plant species of grasslands, belonging to Poaceae and Cyperaceae, are Si accumulators (Conley, 2002; Epstein, 1994; Strömberg et al., 2016) and deposit Si mostly in phytoliths (Hodson et al., 2005; Schaller et al., 2013). In the present study, the highest phytolith content in the aboveground parts of plants was detected in *Carex duriuscula*, a perennial Cyperaceae, whereas the lowest content was in *Salsola collina*, an annual Chenopodiaceae, consistent with results from other studies (Ru et al., 2018). The highest phytolith content in the belowground parts was detected in *Stipa grandis*, a perennial bunchgrass, while the lowest was also in *Salsola collina*. Even though a high Si content is not a general feature of monocots (Hodson et al., 2005), many monocots accumulate more Si than nonmonocots (Epstein, 1994). In the present study, phytolith contents averaged 22.40 and 57.25 g/kg, respectively, in the above‐ and belowground parts of monocots, higher than that in the above‐ and belowground parts of dicots, respectively, 9.14 and 35.88 g/kg. Plant species are distinct in terms of phytolith and PhytOC contents, and the distinction is most likely related with plant phylogeny and their adaptation to environment in early evolutionary time (Epstein, 1994; Li et al., 2014). Poaceae and Cyperaceae are Si‐absorbing plants, that is, they absorb more bioavailable Si from the soil for growth than dicots (Hodson et al., 2005).

Both species composition and NPP of plant communities vary substantially along climatic gradient in semiarid regions (Bai et al., 2004; Dai et al., 2012; Hou et al., 2014), while the biomass‐weighted mean phytolith or PhytOC contents does not show a significant difference among steppe types (Figure 3). This is because that the dominant species in the studied steppe communities at the three sites are phylogenetically close (i.e., belong to the same genera), thus have similar phytolith or PhytOC contents, and contribute to a large proportion of community biomass (Qiu et al., 2016; Ricotta & Moretti, 2011). For example, *Stipa* species are dominants in all the three steppe sites, with their AGB (either of *S. klemenzii, S. grandis,* or *S. krylovii*) contributing to 45.79%, 53.25%, and 48.42% of the community AGB respectively in the desert steppe, the dry typical steppe and the wet typical steppe. Also, dominant *Cleistogenes songorica* or *C. squarrosa* accounts for 15.59%, 9.35%, and 25.04% of the community AGB, respectively, in these three sites. As a result, the variation in community phytolith or PhytOC contents has much smaller effects on its production flux (= phytolith or PhytOC content × NPP) across the steppe sites, and the variation in phytolith or PhytOC production flux is mainly associated with the changes in NPP. This indicates a predominant role played by plant above‐ and belowground biomass production along the climate gradient for the phytolith and PhytOC production, which overshadows the effects of the variation in plant PhytOC content at community level.

The significantly higher PhytOC content in the below‐ than the aboveground biomass is most likely associated with the perenniality of plants. The PhytOC in the aboveground part is deposited in the current year, whereas that in the belowground part may be deposited in multiple years (Qi et al., 2016). The higher PhytOC content in the aboveground part of the desert steppe community than that of the typical steppe communities is consistent with our hypothesis that the intense transpiration required for Si deposition in plant tissues in the desert steppe contributed to the higher PhytOC content (Hattori et al., 2005; Ma & Takahashi, 2002). However, the PhytOC production flux is significantly lower in desert steppe community than the typical steppes due to its lower NPP.

The PhytOC content in plants is associated with the silica deposition, thus with the amount of plant Si uptake (Li, Song, Parr, et al., 2013; Parr et al., 2009). The environment factors, such as climate and soil conditions, may influence plant Si uptake, thus the efficiency of PhytOC accumulation in plants (Li, Song, Parr, et al., 2013; Pan et al., 2017; Yang et al., 2018). In our study, low pH and high SOC can account for the higher phytolith accumulation in the wet than the dry typical steppe, which is consistent with the results in previous report (Song, Wang, et al., 2012). A higher pH and moisture content accelerate Si to dissolve in soil solutions (Fraysse et al., 2009; Yang et al., 2018), and to be taken up by plants and transpired in vascular bundles in plants (Epstein, 2009; Parr et al., 2009). Pan et al. (2017) reports that soil SiO_2_ content shows no significant difference among the grassland sites with different degradation levels, while bioavailable Si content in the top soil of nondegraded grassland was relatively lower due to more bioavailable Si being absorbed by plants at the site. In studied steppes, the bioavailable Si content in the soil was higher in the dry typical steppe site than in other two sites, which is probably resulted from the less uptake of bioavailable Si from the soil at this site, as its phytolith accumulation was lower than in other two steppe sites.

### 4.2 PhytOC long‐term sequestration potential

Many previous studies have assessed the potential of PhytOC sequestration in various ecosystems, such as forest, grassland, wetland, and agriculture ecosystems (Li, Song, Li, et al., 2013; Parr et al., 2009, 2010; Zuo and Lü, 2011). But most of these assessments are based on the PhytOC production in plant aboveground biomass, ignoring that in belowground biomass. A recent study (Chen et al., 2018) reports that the belowground biomass in a bamboo forest accounts for 39.41% of the total plant biomass. Another recent study (Zhang et al., 2019) shows that the litter layer in bamboo forest plays an important role as PhytOC storage. In native steppes, plant aboveground biomass turns to litter in current year, while the belowground biomass is huge and accumulated over multiple years (Chai et al., 2014; Dai et al., 2012; Hou et al., 2014). Qi et al. (2016) showed that PhytOC production from the belowground part is much greater than that from the aboveground part for the typical steppe. The results of the present study show that the predominant role of belowground PhytOC production in phytolith carbon sequestration is mainly a consequence of high BNPP and high accumulated Si content in perennial herbs in permanent grassland (Wiese et al., 2007). This study confirms the previous findings with more data from three steppe types along a precipitation gradient and indicates the necessity to involve the belowground part in the assessment of phytolith carbon sequestration potential.

## 5 CONCLUSIONS

Our study focuses on the phytolith and PhytOC contents of different plant communities and their constituent species on a climate gradient in the semiarid steppe region of the Mongolian Plateau. Our results show that the phytolith and PhytOC contents differ among plant species and that climatic factors affect phytolith and PhytOC production rates of steppe communities mainly through affecting plant production, whereas their effects on the phytolith and PhytOC contents in plants are relatively small. Our results also demonstrate that PhytOC contents in both AGB and BGB had an opposite change trend to that of AGB and BGB, indicating the steppes with low biomass had relatively high PhytOC contents. More studies are warranted to quantify annual PhytOC production from plant belowground part with the effects of plant perenniality incorporated.

## CONFLICT OF INTEREST

None declared.

## AUTHOR CONTRIBUTION

**Limin Qi:** Conceptualization (equal); Data curation (equal); Formal analysis (equal); Investigation (equal); Methodology (equal); Writing‐original draft (equal); Writing‐review & editing (equal). **Tingyu Sun:** Data curation (equal); Formal analysis (equal); Investigation (equal); Writing‐review & editing (equal). **Xudong Guo:** Data curation (equal); Formal analysis (equal); Investigation (equal); Writing‐review & editing (equal). **Ying Guo:** Data curation (equal); Formal analysis (equal); Investigation (equal); Writing‐review & editing (equal). **Frank Yonghong Li:** Conceptualization (equal); Funding acquisition (equal); Investigation (equal); Methodology (equal); Supervision (equal); Writing‐original draft (equal); Writing‐review & editing (equal).

## Data Availability

Data are available from the Dryad Digital Repository: https://orcid.org/0000‐0002‐0932‐391X.
